# Supplementary material for: Discovery and application of insertion-deletion (INDEL) polymorphisms for QTL mapping of early life-history traits in Atlantic salmon
Source: BMC Genomics. 2010 Mar 8;11:156. doi: 10.1186/1471-2164-11-156 (PMC2838853; doi:10.1186/1471-2164-11-156)
Supplement: Additional file 2 — Information on developed 76 locus single-run INDEL panel in Atlantic salmon. Information on fluorescence labeling, primer concentrations, PCR pooling and links to alignments, INDEL motifs and GENESCAN (Burge and Karlin 1997) predictions of genes/exons are available in html format. [file 1471-2164-11-156-S2.ZIP › Additionalfile2/snpsummary7655.html]

```
Cluster 2286 Contig 2

prev  Summary    Contig List  next
```

Size of Consensus sequence = 2705

Number of sequences = 21

Minimum redundancy = 6

Key

A gi|117853522|gb|EG926218.1|EG926218 EST\_ssal\_evf\_28753 ssalevf mixed\_tissue Salmo salar cDNA Salmo salar cDNA clone ssal\_evf\_538\_049\_rev 5', mRNA sequence  
B gi|117493075|gb|EG825292.1|EG825292 EST\_ssal\_eve\_38054 ssaleve thyroid Salmo salar cDNA Salmo salar cDNA clone ssal\_eve\_551\_311\_rev 5', mRNA sequence  
C gi|24394614|gb|CA064371.1|CA064371 ssalrgb501298 mixed\_tissue Salmo salar cDNA, mRNA sequence  
D gi|85015495|gb|DW544151.1|DW544151 EST\_ssal\_rgb2\_8570 rgb2 Salmo salar cDNA clone ssal\_rgb2\_514\_335\_fwd 3', mRNA sequence  
E gi|85017116|gb|DW545772.1|DW545772 EST\_ssal\_rgb2\_10191 rgb2 Salmo salar cDNA clone ssal\_rgb2\_517\_144\_fwd 3', mRNA sequence  
F gi|117458043|gb|EG790262.1|EG790262 EST\_ssal\_evd\_10556 ssalevd thymus Salmo salar cDNA Salmo salar cDNA clone ssal\_evd\_512\_352\_rev 5', mRNA sequence  
G gi|117493842|gb|EG826059.1|EG826059 EST\_ssal\_eve\_38745 ssaleve thyroid Salmo salar cDNA Salmo salar cDNA clone ssal\_eve\_552\_284\_rev 5', mRNA sequence  
H gi|29320878|gb|CB509652.1|CB509652 ssalnwh503360 whole Salmo salar cDNA, mRNA sequence  
I gi|117493076|gb|EG825293.1|EG825293 EST\_ssal\_eve\_38055 ssaleve thyroid Salmo salar cDNA Salmo salar cDNA clone ssal\_eve\_551\_311\_fwd 3', mRNA sequence  
J gi|117829740|gb|EG902436.1|EG902436 EST\_ssal\_evf\_41094 ssalevf mixed\_tissue Salmo salar cDNA Salmo salar cDNA clone ssal\_evf\_554\_328\_rev 5', mRNA sequence  
K gi|117829739|gb|EG902435.1|EG902435 EST\_ssal\_evf\_41093 ssalevf mixed\_tissue Salmo salar cDNA Salmo salar cDNA clone ssal\_evf\_554\_328\_fwd 3', mRNA sequence  
L gi|117493843|gb|EG826060.1|EG826060 EST\_ssal\_eve\_38746 ssaleve thyroid Salmo salar cDNA Salmo salar cDNA clone ssal\_eve\_552\_284\_fwd 3', mRNA sequence  
M gi|117458044|gb|EG790263.1|EG790263 EST\_ssal\_evd\_10557 ssalevd thymus Salmo salar cDNA Salmo salar cDNA clone ssal\_evd\_512\_352\_fwd 3', mRNA sequence  
N gi|117853521|gb|EG926217.1|EG926217 EST\_ssal\_evf\_28752 ssalevf mixed\_tissue Salmo salar cDNA Salmo salar cDNA clone ssal\_evf\_538\_049\_fwd 3', mRNA sequence  
O gi|117299337|gb|EG649149.1|EG649149 SGP261505 Atlantic salmon normalized Muscle cDNA library Salmo salar cDNA clone NM1-1448 5', mRNA sequence  
P gi|85015494|gb|DW544150.1|DW544150 EST\_ssal\_rgb2\_8569 rgb2 Salmo salar cDNA clone ssal\_rgb2\_514\_335\_rev 5', mRNA sequence  
Q gi|85047723|gb|DW575901.1|DW575901 EST\_ssal\_rgb2\_40320 rgb2 Salmo salar cDNA clone ssal\_rgb2\_565\_196\_rev 5', mRNA sequence  
R gi|45304332|gb|CK874701.1|CK874701 SGP135917 Atlantic salmon Brain cDNA library Salmo salar cDNA clone HJ4-0834 5', mRNA sequence  
S gi|29328967|gb|CB517741.1|CB517741 ssalrgb501298\_rev mixed\_tissue Salmo salar cDNA, mRNA sequence  
T gi|85017115|gb|DW545771.1|DW545771 EST\_ssal\_rgb2\_10190 rgb2 Salmo salar cDNA clone ssal\_rgb2\_517\_144\_rev 5', mRNA sequence  
U gi|85050239|gb|DW578417.1|DW578417 EST\_ssal\_rgb2\_42836 rgb2 Salmo salar cDNA clone ssal\_rgb2\_569\_167\_rev 5', mRNA sequence

2 SNPs detected

A B C D E F G H I J K L M N O P Q R S T U  cosegregation weighted

957 . . . - T T T - - - - T T - - T . . . . .   2/2 61.90
958 . . . - T T T - - - - T T - - T . . . . .   2/2 61.90
